# Appropriate feeding practice and associated factors among under-five children with diarrheal disease in sub-Saharan Africa: a multi-country analysis

**DOI:** 10.1186/s41182-023-00503-1

**Published:** 2023-03-01

**Authors:** Yigizie Yeshaw, Adugnaw Zeleke Alem, Hiwotie Getaneh Ayalew, Alemneh Mekuriaw Liyew, Zemenu Tadesse Tessema, Misganaw Gebrie Worku, Getayeneh Antehunegn Tesema, Tesfa Sewunet Alamneh, Achamyeleh Birhanu Teshale

**Affiliations:** 1 Department of Physiology, School of Medicine, College of Medicine and Health Sciences, University of Gondar, P. O. Box 196, Gondar, Ethiopia; 2 Department of Epidemiology and Biostatistics, Institute of Public Health, College of Medicine and Health Sciences, University of Gondar, P. O. Box 196, Gondar, Ethiopia; 3 Department of Midwifery, School of Nursing and Midwifery, College of Medicine and Health Sciences, Wollo University, Dessie, Ethiopia; 4 Department of Human Anatomy, School of Medicine, College of Medicine and Health Sciences, University of Gondar, P. O. Box 196, Gondar, Ethiopia

**Keywords:** Appropriate feeding practice, Diarrhea, Children, Sub-Saharan Africa

## Abstract

**Background:**

Diarrheal disease is one of the leading causes of child mortality and morbidity in low-income countries. Although the provision of more fluid and solid foods during diarrhea are important to treat the diseases, in Africa, food and fluid restrictions are common during diarrheal illness. Therefore, the aim of this study was to determine appropriate feeding practice and associated factors among under-five children with diarrheal disease in sub-Saharan Africa (SSA).

**Methods:**

We have used the appended most recent demographic and health survey (DHS) datasets of 35 sub-Saharan countries conducted from 2010 to 2020. A total weighted sample of 42,882 living children with diarrhea were included in the analyses. Multivariable multilevel binary logistic regression was used to identify factors associated with appropriate child feeding practice in SSA. A *p* value of ≤ 0.05 was used as a cut of point to declare statistically significant variables.

**Results:**

The overall prevalence of appropriate child feeding practice in this study was 10.45% (95% CI 10.17–10.74). The odds of having appropriate child feeding practice was higher among women with primary (AOR = 1.27: 1.17–1.37), secondary (AOR = 1.38: 1.25–1.52), and higher education level (AOR = 1.52: 1.21–1.90), media exposure (AOR = 1.11: 1.11–1.29), richer (AOR = 1.23:1.01–1.26) and richest (AOR = 1.19:1.05–1.35) wealth index, and currently working (AOR = 1.12: 1.04–1.19).

**Conclusion:**

The prevalence of appropriate child feeding practice in this study was found to be very low. It advisable to reduce diarrhea-related child mortality through enhancing diarrhea management practice especially by working on the after mentioned factors.

## Background

Diarrheal disease is one of the leading causes of child mortality and morbidity in the world [1, 2]. Although diarrhea is present globally among all regions and populations, an inequitable proportion of diarrhea morbidity and mortality occurs in low-income countries, areas where fewer resources and less robust infrastructure exist [3]. It was responsible for an estimated 533,768 deaths of children younger than 5 years globally in 2017 [4]. It accounts for one in eight deaths among children younger than 5 years per annum in Africa, Asia, and South America [5, 6].

If not appropriately managed, childhood diarrhea can cause impaired uptake of macronutrients and micronutrients, further episodes of infectious diseases, malnutrition, impaired physical growth, and cognitive development [7, 8]. Optimal child feeding practices (both liquid and solid foods) during childhood illnesses are among the foremost effective global strategies of integrated management of childhood illnesses and emphasize the necessity to extend fluid intake during illness while feeding is maintained during convalescence [9–11].

Continuing feeding during diarrheal episode can lessen the consequences of diarrheal disease as, thereby the death of child can be prevented [12–14].

The finding of previous studies revealed that occupation [15], wealth index [16], maternal age [12, 15], maternal education [16, 17], number of under-five children, and knowledge on child feeding practice [12, 18] are associated with appropriate child feeding practice.

Although the provision of more fluid and solid foods during diarrhea is important to treat the diseases [1], in Africa, food and fluid restrictions during diarrhea are common [17, 19] and only 35% of under-five children with diarrhea get appropriate fluid replacement during diarrheal episodes in the region [20]. Therefore, the main aim of this study was to determine appropriate feeding practice and associated factors among under-five children with diarrheal disease in sub-Saharan Africa.

## Methods

### Data source

We have used the appended demographic and health survey (DHS) datasets of 35 sub-Saharan countries, conducted from 2010 to 2020, to determine the prevalence and associated factors of appropriate child feeding practice during diarrhea illness in the region. The DHS used pretested standard questionnaires to collect data on population and health indicators every 5 years. The datasets of these countries were obtained from DHS Program website, after reasonable request. All under-five living children who had diarrhea 2 weeks preceding the survey were included in this analysis.

### Variables of the study

The outcome variable for this study was appropriate child feeding practice. To determine the outcome, the respondents were asked how much fluid was given to drink for their child during the diarrhea and they respond as less than usual to drink, about the same amount, or more than usual to drink. Similarly, for food, they were asked how much food was given to eat for their child during the diarrhea and they respond as less than usual to eat, about the same amount, or more than usual to eat [21]. Accordingly, in this study, those children with diarrhea who were given more liquids than usual, and as much food or more than usual were said to have appropriate child feeding practice [14, 22].

The independent variables for this study include both individual and community-level factors. These were marital status, education level of husband, wealth index, education level of mother, residence, perception of distance from health facility, sex of household head, sub-Saharan Africa region (west Africa, east Africa, central Africa, and western Africa), age of household head, current working status of women (yes or no), and media exposure (a composite variable of listing radio, watching television, and reading newspaper, in which women were said to have media exposure “if they have exposed to either of these media sources” and no “if did not have exposure to all of the above media sources”).

### Data analysis procedure

STATA 14 software was used analyze the data. As to the recommendation DHS statistics guide [21], the data were weighted before doing any statistical analysis to restore the representativeness and get a reliable standard error.

Considering the hierarchical nature of DHS data, different measures of community variations, such as Median Odds Ratio (MOR), Intraclass Correlation Coefficient (ICC), and Proportional Change in Variance (PCV), were estimated. Based on the values of these measures, multilevel logistic regression model was used. To choose the most appropriate model, four models were fitted (null model to model three) and then these models were compared using deviance. These were the null model (a model with no independent variable), model I (a model with individual-level factors only), model II (a model with community-level factors only), and model III (a model that contain both individual and community-level variables). Of the four models, a model with the lowest deviance was selected as best fitted model (model III).

Just after selecting the most appropriate model for the data, both bivariable and multivariable multilevel binary logistic regression were performed to identify the associated factors of appropriate feeding practice of under-five children during diarrheal episode in SSA. In the bivariable analysis, those variables with a *p* value < 0.25 were selected as candidate variables for the multivariable multilevel binary logistic regression model. A *p* value ≤ 0.05 was used to declare statistically significant variables.

## Results

### Characteristics of study participants

A total weighted samples of 42,882 living children with diarrhea were included in these analyses. More than two-third, 29,561 (68.94%), of the participants were rural dwellers. Majority, 36,357 (84.78%), of them were currently married. Twenty-six thousand and seven hundred eighty four (62.46%) of the participants were currently working. Most of the participants had media exposure (65%) and not perceive distance from health facility as big problem (61.19%) (Table 1).

**Table 1:** Socio demographic characteristics of respondents

Variables	Weighted frequency	Percentage
Residence		
Urban	13,321	31.06
Rural	29,561	68.94
Marital status		
Never married	3145	7.33
Married	36,357	84.78
Formerly married	3380	7.88
Currently working		
Yes	26,784	62.46
No	16,097	37.54
Education level of mother		
No education	15,593	36.36
Primary education	16,109	37.57
Secondary education	10,167	23.71
Higher education	1013	2.36
Perception of distance from health facility		
No big problem	26,240	61.19
Big problem	16,641	38.81
Wealth index		
Poorest	9953	23.21
Poorer	9620	22.43
Middle	8526	19.88
Richer	8172	19.06
Richest	6611	15.42
Sex of household head		
Male	33,822	78.87
Female	9060	21.13
Media exposure		
Yes	27,875	65.00
No	15,007	35.00
Education level of husband		
No education	19,491	45.45
Primary education	11,108	25.90
Secondary education	10,193	23.77
Higher education	2089	4.87

### Random-effects analysis

The result of random-effects analysis below shows the presence of significant clustering of appropriate feeding practice. Both the community-level variance and the MOR value of null model were significant indicating the presence of community-level variability of appropriate child feeding practice during diarrheal episode. The MOR of the null model indicates women who are found at a cluster with higher appropriate feeding practice had 1.32 times higher odds of having appropriate child feeding practice compared to their counterparts. The PCV value increases from null model to model III, and hence, the last model better explains the variability of appropriate feeding practice. Moreover, model III has the lowest Deviance value. As result, it was selected as the best fitted model (Table 2).

**Table 2:** Result of model comparison and random-effects analysis

Parameters	Null model	Model I	Model II	Model III
Community variance	0.085 (0.060–0.121)	0.084 (0.059- 0.119)	0.084 (0.059–0.119)	0.082 (0.057- 0.117)
ICC	2.53% (1.80–3.54)	2.49 (1.76- 3.50)	2.48% (1.76–3.49)	2.42% (1.70- 3.43)
MOR	1.32 (1.26–1.39)	1.31	1.31	1.31
PCV	Reference	1.76%	1.87%	4.33%
Model fitness				
Deviance (-2 LL)	28,291.44	28,098.738	28,261.862	28,087.446

### Prevalence of appropriate child feeding practice

The overall prevalence of appropriate child feeding practice in this study was 10.45% (95% CI 10.17–10.74). About one-fourth of children (24.53%) were given more liquids than usual and 17,466 (40.73%) were given as much food or more than usual. Only, 2052 (4.78%) were given both more fluid and food than usual.

### Associated factors of appropriate child feeding practice

On the bivariable multilevel binary logistic regression analysis, residence, perception of distance to the health facility, media exposure, wealth index, education level of the mother, education level of husband, and current working status of the mother were associated with appropriate child feeding practice (*p* ≤ 0.25). In the multivariable multilevel binary logistic regression analysis, wealth index, media exposure, current working status of mother, and education level of the mother were found to be significantly associated with appropriate child feeding practice (*p* ≤ 0.05).

The odds of having appropriate child feeding practice was higher among those women with primary (AOR = 1.27: 1.17–1.37), secondary (AOR = 1.38: 1.25–1.52), and higher education level (AOR = 1.52: 1.21–1.90) than those with no education. Those women with media exposure had 1.11 (AOR = 1.11: 1.11–1.29) times higher chance of having appropriate child feeding practice than their counterparts. Women’s of richer (AOR = 1.23:1.01–1.26) and richest (AOR = 1.19:1.05–1.35) wealth index had higher odds of having a child with appropriate feeding practice during diarrheal episode compared to women of poorest wealth index. Those women with current work had 1.12 (AOR = 1.12: 1.04–1.19) times higher chance of having appropriate child feeding practice than their counterparts (Table 3).

**Table 3:** Multilevel logistic regression analyses for the factors associated with appropriate feeding practice in sub-Saharan Africa

Variables	Odds ratio	
	COR (95% CI)	AOR (95% CI)
Residence		
Urban	1.21 (1.13–1.29)	1.001 (0.92–1.09)
Rural	1	1
Education level of mother		
No education	1	1
Primary education	1.35 (1.25–1.45)	1.27 (1.17–1.37)*
Secondary education	1.56 (1.43–1.69)	1.38 (1.25–1.52)*
Higher education	1.81 (1.48–2.22)	1.52 (1.21–1.90)*
Wealth index		
Poorest	1	1
Poorer	1.11 (1.01–1.22)	1.04 (0.95–1.15)
Middle	1.17 (1.06–1.29)	1.06 (0.96–1.18)
Richer	1.29 (1.17–1.42)	1.23 (1.01–1.26)*
Richest	1.46 (1.32–1.61)	1.19 (1.05–1.35)*
Perception of distance to health facility		
Not big problem	1.06 (1.01–1.13)	0.99 (0.93–1.06)
Big problem	1	1
Women currently working		
Yes	1.11 (1.04–1.19)	1.12 (1.04–1.19)*
No	1	1
Husband education level		
No education	1	1
Primary education	1.15 (1.07–1.25)	1.06 (0.98–1.15)
Secondary education	1.29 (1.19–1.39)	1.08 (0.99–1.17)
Higher education	1.37 (1.19–1.59)	1.01 (0.86–1.18)
Media exposure		
Yes	1.34 (1.25–1.44)	1.20 (1.11–1.29)*
No	1	1

*p ≤ 0.05

## Discussion

Inappropriate management of diarrhea can result in an increased risk of mortality through dehydration or long-lasting health consequences [19]. Therefore, investigating the magnitude of appropriate feeding practice and associated factors among under-five children with diarrheal disease is crucial to tailor different intervention measures for the reduction of diarrhea-related mortality among under-five children in sub-Saharan Africa.

In this study, only 10.45% of under-five children with diarrhea had appropriate feeding during diarrheal episode. As the figure reveals that, a large number of children are not receiving adequate food and fluid during their diarrheal episode in the region indicating the issue an urgent priority [23].

Regarding associated factors, in this study, wealth index, education level of mother, media exposure, and working status of mother significantly affect appropriate child feeding practice.

In line with studies conducted elsewhere [16, 17, 24], in this study, the odds of having appropriate child feeding practice was higher among educated women compared to none educated once. This could be due to the increase in mother’s ability to take care of their child in appropriate way following education. This is because education increases the knowledge of women about the management of diarrhea [24–26].

Those women with media exposure had an increased chance of having appropriate child feeding practice than their counterparts. This finding is in line with other studies [27–30]. The possible reason is that mass media have the potential to increase the knowledge and attitudes of the women by delivering health messages [31–33]. Hence, both knowledge and the use of fluid and food as management of diarrhea are higher among mothers with media exposure [34]. This indicates the need to increase media exposure for mothers to increase child survival in low-income countries [35].

Consistent with other studies [16, 36], in this study, women’s of richer and richest wealth index had higher odds of having a child with appropriate feeding practice compared to women of poorest wealth index. This is obviously because women with high income have no budget constraints to buy different food items and fluids to use for the treatment of diarrhea compared to their counterparts.

Another factor that is significantly associated with appropriate child feeding practice in this study was working status of women in that those women with current work had higher chance of having appropriate child feeding practice than their counterparts [37]. This could be due to the positive impact of women employment on household wealth status in that those working women usually have financial resources that enable them to purchase adequate food and feed for their child.

Our study has its own strengths and limitations. First of all, to the best of our knowledge, this study is the first in determining the prevalence and associated factors of appropriate child feeding practice among under-five children in SSA. The use of large sample size enhances the generalizability of this study. Moreover, advanced statistical analysis was performed that enable to yield reliable standard errors. Regarding limitation, since the data were collected based on mother’s recall, there may be the possibility of recall bias.

## Conclusion

The prevalence of appropriate child feeding practice in this study was low. Wealth index, media exposure, working status, and education level of the women were significantly associated with appropriate child feeding practice. The finding of this study reveals the need to enhance diarrhea management practice and reduce diarrhea-related mortality, preventable cause of child death, in the region.

## Data Availability

All relevant data related to the study were included in this manuscript. The datasets used for the analysis of the study can also be obtained after reasonable request of the DHS Program using the link https://dhsprogram.com/data/dataset_admin/index.cfm.

## Abbreviations

AOR: Adjusted Odds Ratio
DHS: Demographic and Health Survey
CI: Confidence interval
COR: Crude Odds Ratio
ICC: Intraclass Correlation Coefficient
MOR: Median Odds Ratio
PCV: Proportional Change in Variance
SSA: Sub-Saharan Africa
